# Community Resilience throughout the Lifespan – The Potential Contribution of Healthy Elders

**DOI:** 10.1371/journal.pone.0148125

**Published:** 2016-02-04

**Authors:** Odeya Cohen, Diklah Geva, Mooli Lahad, Arkady Bolotin, Dima Leykin, Avishay Goldberg, Limor Aharonson-Daniel

**Affiliations:** 1 Department of Emergency Medicine, Recanati School for Community Health Professions, Faculty of Health Sciences, Ben-Gurion University of the Negev, Beer-Sheva, Israel; 2 PREPARED Center for Emergency Response Research, Ben-Gurion University of the Negev, Beer-Sheva, Israel; 3 Department of Epidemiology, Faculty of Health Sciences, Ben-Gurion University of the Negev, Beer-Sheva, Israel; 4 Department of Psychology, Tel-Hai Academic College, Kiryat-Shmona, Israel; 5 The Community Stress Prevention Centre (CSPC), Kiryat-Shmona, Israel; 6 Department of Public Health, Faculty of Health Sciences, Ben-Gurion University of the Negev, Beer-Sheva, Israel; 7 Department of Health Systems Management, Faculty of Health Sciences & Guilford Glazer Faculty of Business and Management, Ben-Gurion University of the Negev, Beer-Sheva, Israel; Yokohama City University, JAPAN

## Abstract

An increase in the exposure and predisposition of civilian populations to disasters has been recorded in the last decades. In major disasters, as demonstrated recently in Nepal (2015) and previously in Haiti (2010), external aid is vital, yet in the first hours after a disaster, communities must usually cope alone with the challenge of providing emergent lifesaving care. Communities therefore need to be prepared to handle emergency situations. Mapping the needs of the populations within their purview is a trying task for decision makers and community leaders. In this context, the elderly are traditionally treated as a susceptible population with special needs. The current study aimed to explore variations in the level of community resilience along the lifespan. The study was conducted in nine small to mid-size towns in Israel between August and November 2011 (N = 885). The Conjoint Community Resiliency Assessment Measure (CCRAM), a validated instrument for community resilience assessment, was used to examine the association between age and community resilience score. Statistical analysis included spline and logistic regression models that explored community resiliency over the lifespan in a way that allowed flexible modeling of the curve without prior constraints. This innovative statistical approach facilitated identification of the ages at which trend changes occurred. The study found a significant rise in community resiliency scores in the age groups of 61–75 years as compared with younger age bands, suggesting that older people in good health may contribute positively to building community resiliency for crisis. Rather than focusing on the growing medical needs and years of dependency associated with increased life expectancy and the resulting climb in the proportion of elders in the population, this paper proposes that active "young at heart" older people can be a valuable resource for their community.

## Introduction

Community resilience reflects the community's capacity to overcome changes and crises. The development and enhancement of community resilience during the pre-emergency period can serve as a core capability of communities in emergency situations [1]. Community resilience has also been related to sustainable lifestyle [2], [3], and a provider of the society's adaptive capacities during crisis situations [4], [5]. The term ‘community resilience’ describes a complex construct that encompasses social aspects such as leadership, collective efficacy, social cohesion and place attachment, along with physical dimensions such as infrastructure, services and protection [1], [6]–[8]. It is discussed in the literature in many disciplines, content fields and levels, resulting in a multiplicity of definitions that reflect the abundance of perspectives. However, we have chosen to comply with the functional definition phrased above.

There is a close relationship between the medical context, or public health, and community resiliency [9], [10]. Castleden et al. [11] claim that a healthy population is one of the significant rewards of promoting community resilience. Chandra et al. [12] found that community resilience is connected to state of physical and mental health in the pre-emergency period, among other elements. Other aspects are related to the continuity of health services during the different phases of the emergency situation and to the ability to share updated medical information during the time of change [10], [12]. Poortinga [13] found that attributes of community resilience were significantly associated with self-reported health.

An increase in the exposure of civilian populations to disasters has been recorded in the last decades. In major disasters, as demonstrated recently in Nepal (2015) and previously in Haiti (2010), external aid is vital, yet in the first hours after a disaster, communities must usually cope alone with the challenge of providing emergent lifesaving care.

Within the overlapping worlds of disaster preparedness, resiliency and health, special attention should be paid to the issue of the aging population. The percentage of older adults in the general population is increasing, and it is important to assess and attend to the consequences. According to Wild et al. [14], most studies dealing with resilience late in life fail to examine the role of the community. In those studies that do consider the community, researchers analyze its role only in terms of the impact that aging has on individual resilience. Wiles et al. [15] note that, especially in the later stage of life, the frame of reference of resiliency expands, encompassing both the resources of the community and individual aspects of resiliency. From such a perspective an older person with a major illness or hardship could be perceived as “aging well” or even as resilient.

There are different approaches regarding the ageing population during situations of change [16]. On the one hand, the elder population is perceived as vulnerable [12], [17]–[19]. The CDC reports that disasters of all kinds affect older adults, especially those with severe chronic diseases [20], and other studies assign resilient attributes to neighborhoods with low percentages of elder residents [21]. On the other hand, there is a different trend focused on the positive influence of elders on their community. Wiles and Jayasinha [22] found that older people care about their place of residence, becoming involved in volunteering, activism, advocacy, and nurturing others in the community. Similar findings emerged in the work of Alessa et al. [23], [24], showing that the presence of elderly members strengthens the community’s resiliency to cope with changes. According to Kimhi et al. [25], [26], older individuals who had been exposed to security threats showed a higher level of community resilience as compared with younger participants.

In general, the literature reveals a dearth of empiric evidence from community resilience studies [12], [27]. The complexity of the term ‘community resilience’ and the diversity of content worlds that use the term make it difficult to measure this attribute or to aggregate empirical findings in agreed research frameworks [11]. This state of affairs becomes particularly acute in the case of research on resiliency among ageing populations [15], [16].

The state of community resilience research was the driving force behind the development of the Conjoint Community Resilience Assessment Measurement (CCRAM), a tool for assessing community resilience. This tool was developed and validated by a group of multidimensional experts and reflects the integration of their knowledge and experience [6], [28]. Several large studies have been conducted using CCRAM mapping of resiliency in various communities with a view to exploring their weaknesses and strengths in both routine and crisis situations. These are described elsewhere [6], [28], [29].

Given the lack of empirical evidence about community resilience in later life and the plethora of contradictory views regarding the capacities of the ageing population in the face of change, the aim of the current paper was to present trends in the level of community resilience along the lifespan, as portrayed by a population based study of this attribute.

## Materials and Methods

Community resilience was assessed using the CCRAM tool [28]. The instrument includes 22 background questions followed by 28 items on a 5-point Likert scale (1—strongly disagree to 5—strongly agree) regarding various aspects of the community and social life of the responders. These items were shown in previous studies [6] to form constructs of five factors: leadership, collective efficacy, preparedness, place attachment, and social trust. The CCRAM score is composed of the average score of the constructs of these factors, each being assigned equal weight.

The study was conducted in nine small to mid-size towns (up to 50,000 inhabitants) in Israel from August to November 2011. The size of the community was found to be significantly associated with community resilience scores, the small communities (up to 10,000 inhabitants) being characterized by higher levels of communal features than mid-size cities [28]. The study used both door to door surveys of randomly selected addresses, and electronic questionnaires distributed to mailing lists in small communities for which a full list of residents was available. The study was approved by the institutional review board (IRB) of the Faculty of Health Sciences at Ben-Gurion University of the Negev. Participants gave their informed consent to take part in the study. A brief introduction at the beginning of the questionnaire described the objectives of the study and specified that filling the questionnaire was voluntary and could be terminated at any time, and also that the questionnaires were anonymous. Continuing to answer the questions represented informed consent, as approved by the IRB.

### Statistical analysis

The study used several statistical approaches to measure the variance of the CCRAM scores over the age distribution. Non-linear regression with spline was used to explore the relationship between CCRAM score and age. In this analysis we fit the knots signifying the points where the linear slope changed [30]. Additional preliminary analysis examined the relationship between the CCRAM score and other study covariates using correlation coefficient analysis and chi-square tests. In order to compare the values of the overall CCRAM scores and the scores for its factors over different ages, the study used five age categories: A—< 30 years, n = 179; B—31–45 years, n = 290; C—46–60 years, n = 249; D—61–75 years, n = 136; E—> 75 years, n = 23. The effect of age group on the community resilience factors–leadership, collective efficacy, preparedness, place attachment and social trust–was examined using multivariate analysis of variance (MANOVA). A logistic regression model was used to explore the characteristics of high average CCRAM scores (in the range of 4–5, n = 245) versus low average CCRAM scores (scores in the range of 1–2.99, n = 205). ANOVA with Scheffe post-hoc test confirmed significant differences among these levels: (*F*(2,882) = 1928.91.91, *p* < 0.001). (Cases associated with an intermediate level of average CCRAM score–scores in the range 3–3.99, n = 433 –were omitted from this analysis.) The first logistic regression analysis modeled age for the five categories mentioned above, with ages 31–45 taken as the reference. The second model forced the previously identified covariates that were self-reported by the responders: gender, marital status, children at home, physical or mental disability that may hamper the responder during emergencies, religion, travel time to work, education level, income level, belonging to a Community Emergency Response Team (CERT), and community type. For all logistic regression models, the odds ratios (OR) along with 95% confidence interval (95% CI) were reported. Sensitivity analysis for selected covariates was used in order to demonstrate graphically the trends of the CCRAM score for subgroups across age categories in a multi-panel figure. Finally, the characteristics of the five age brackets with descriptive statistics (mean±SE) and Bonferoni corrected post hoc pairwise comparisons were detailed. All other p-values reported at significance level of p = 0.05 with no correction for multiple testing. Data were analyzed using Statistical Package for the Social Sciences (SPSS) version 18.0.

## Results

This study sampled adults (N = 885) from nine small to mid-size towns in Israel: mid-size towns (n = 465) and small communities (n = 417). Response rate ranged from 80–95% in the different types of communities, with the smaller communities showing the higher response rates.

The mean age of responders was 45.28 years (median = 44, range 18–85 years, SD = 15.40 years). A significant difference was found between community types in responders’ ages (*t*(765.61) = -4.45, *p* < 0.001): responders living in small communities were older on average (mean age = 47.94, SD = 16.22) than those living in mid-size cities (mean age = 43.25, SD = 14.33). On average responders lived in the same community for 27.42 years (range 1–67 years, SD = 16.9 years). Among the participants, 55.7% were women (n = 490), and 70.5% were in a permanent relationship (n = 625). Forty two percent had an academic degree (n = 368) and 34.8% reported their income as similar to the average income level (n = 308). With regard to disability, 14.6% of the responders mentioned that they had a physical or mental disability which might hamper their functionality in an emergency situation (n = 128). The claim for a disability was significantly higher in the two older age groups above age 60 (*χ*2 (*df* = 4, n = 871) = 128.75, p < 0.01).

The CCRAM average score was 3.49 (1.43–5 range, SD = 0.711), with no significant difference between genders and reported income levels according to the analysis of variance (S1 and S2 Tables).

Pearson correlation analysis detected a significant weak association between CCRAM scores and age, r = 0.187, at the significance level p < 0.001. Further, age was found to be associated with leadership, r = 0.161, p < 0.001, preparedness, r = 0.131, p < 0.001, and place attachment, r = 0.238, p < 0.001; collective efficacy and social trust were not found to be significantly correlated with age.

**Fig 1:**
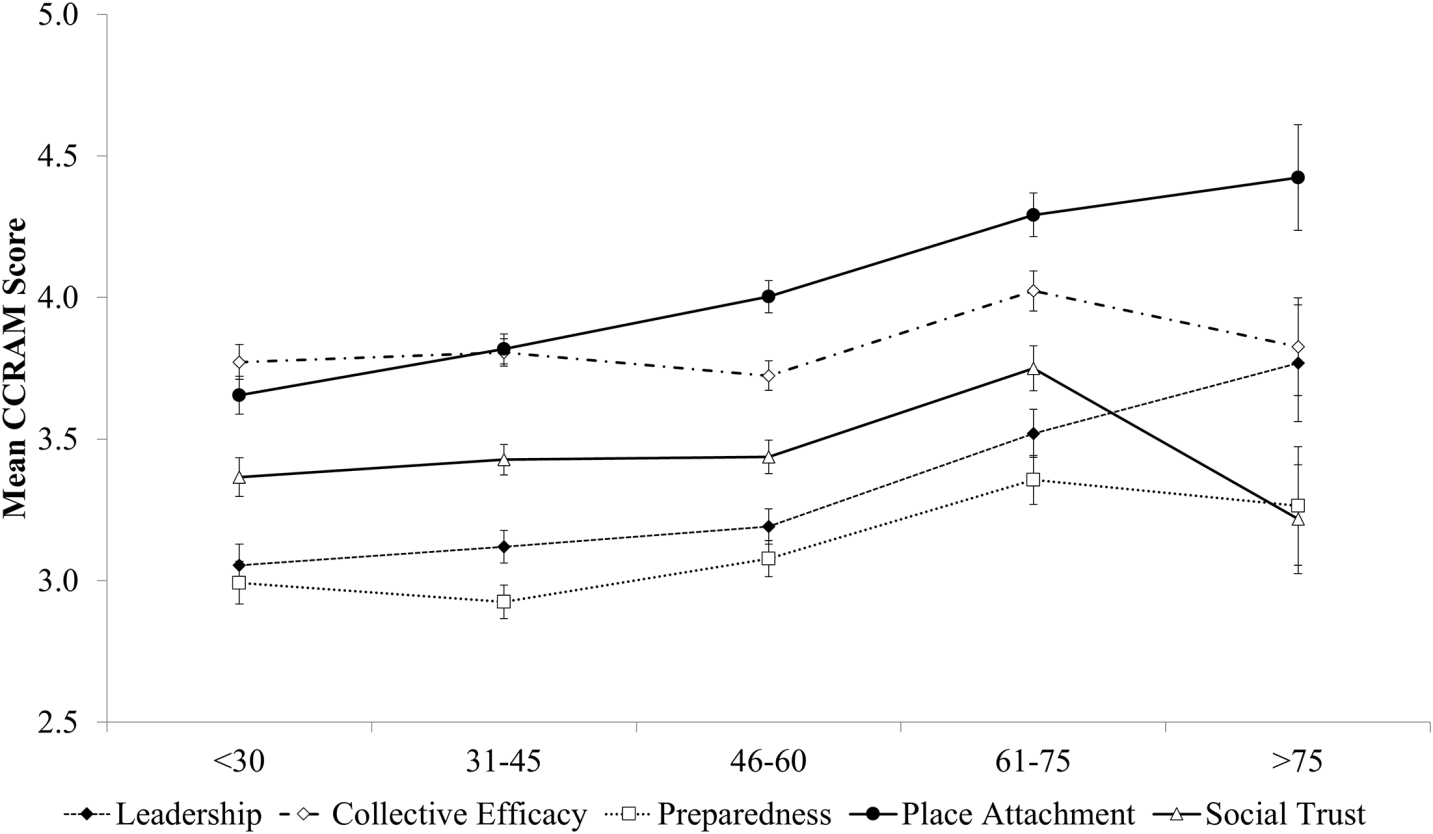
Mean CCRAM scores by age. Notes: spline knots occur at age 27, 52, 72 and 83. Ages over 75 were characterized by scarce and heterogeneous data (n = 23).

Fig 1 shows the mean CCRAM score over age overlaid with a spline fitted curve. The fitted spline curve was statistically significant with F(5,876) = 9.162, p < 0.001, with 4 knots at ages 27, 52, 72 and 83 years. At each knot the slope, b, takes a significant turn; at age 27 b = 0.049 (p = 0.011), at age 52 b = 0.016 9 (p = 0.045), at age 72 b = -0.065 (p = 0.016), and at age 83 b = 0.587 (p = 0.031). This means that CCRAM increases with age during most of adult life and peaks at the age of 72. At the late age of 83 years, we noticed a sharp increase in the CCRAM score with age. However, this late life trend is uncertain, as the group of participants over the age of 75 years was small (n = 23) and heterogeneous due to varied demographics and health characteristics.

**Fig 2:**
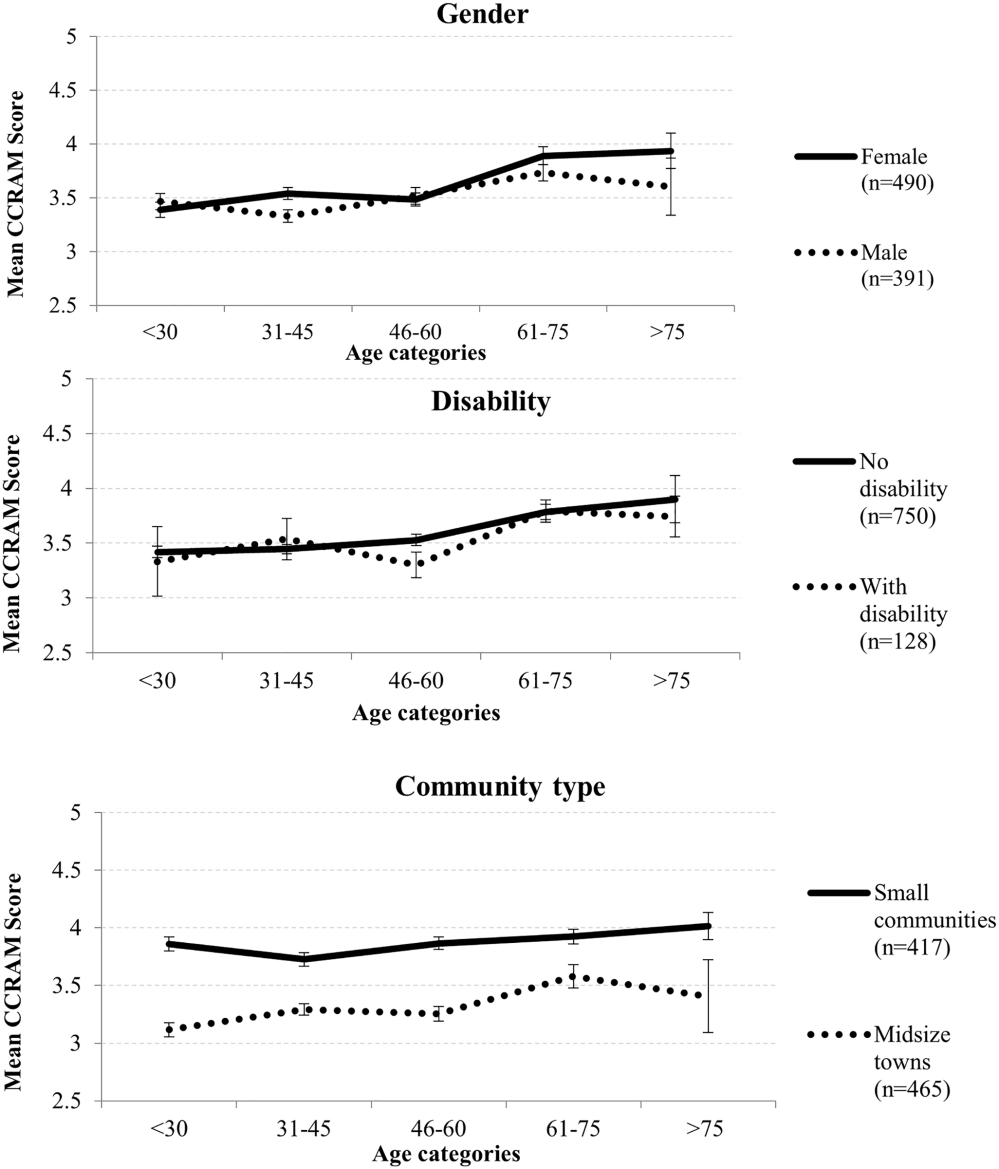
CCRAM factors according to age categories.

At the second stage the mean scores of the CCRAM factors were examined over age. Fig 2 presents the CCRAM factor mean scores according to age categories.

MANOVA was used in order to examine the effect of age group on community resilience factors. This analysis yielded a significant multivariate effect for age group, *F*(20, 3480) = 4.84, *p* < .001, η_p_^2^ = .027. Univariate effects of the resilience factor resulted in significant effects for all factors (see Table 1).

**Table 1:** Average scores for five community resilience factors over age groups.

							Age Group				
Factor	<30(n = 179)		31–45(n = 290)		46–60(n = 249)		61–75(n = 136)		>75(n = 23)		*F*
	*M*	*SD*	*M*	*SD*	*M*	*SD*	*M*	*SD*	*M*	*SD*	
Leadership	3.05	.90	3.12	.80	3.19	1.00	3.52^A^^B^	1.05	3.77	.97	6.91***
Collective Efficacy	3.77	1.00	3.81	.81	3.72	.96	4.02^C^	.94	3.83	.93	3.03*
Preparedness	2.99	1.07	2.93	.86	3.08	1.04	3.36^A^^B^	.85	3.26	.92	4.73***
Place Attachment	3.66	.94	3.82	.81	4.00	1.01	4.29^A^^B^^C^	.68	4.42	.83	13.07***
Social Trust	3.37	.85	3.43	1.00	3.44	1.15	3.75^A^^B^^C^	.60	3.22	1.03	4.35**

Note: df = 4, df error = 871.

* p < .05;

** p < .01;

*** p < .001.

^A^ = <30 years,

^B^ = 31–45 years,

^C^ = 46–60 years.

As detailed in Table 1, post-hoc multiple comparison using Tukey HSD yielded the following results: For the factor of leadership, the 75+ age group differed significantly from the <30 and 31–45 age groups, but not significantly from the 46–60 and 61–75 age groups. For the factor of collective efficacy, the only significant difference was found between the 46–60 and 61–75 age groups (*p* < .001). For preparedness, the 61–75 age group scored significantly higher for this factor compared to the <30 and 31–45 age groups. For place attachment, the 61–75 age group differed significantly from the younger age groups but not from the 75 and above age group. A similar pattern was observed for the social trust factor.

**Fig 3:**
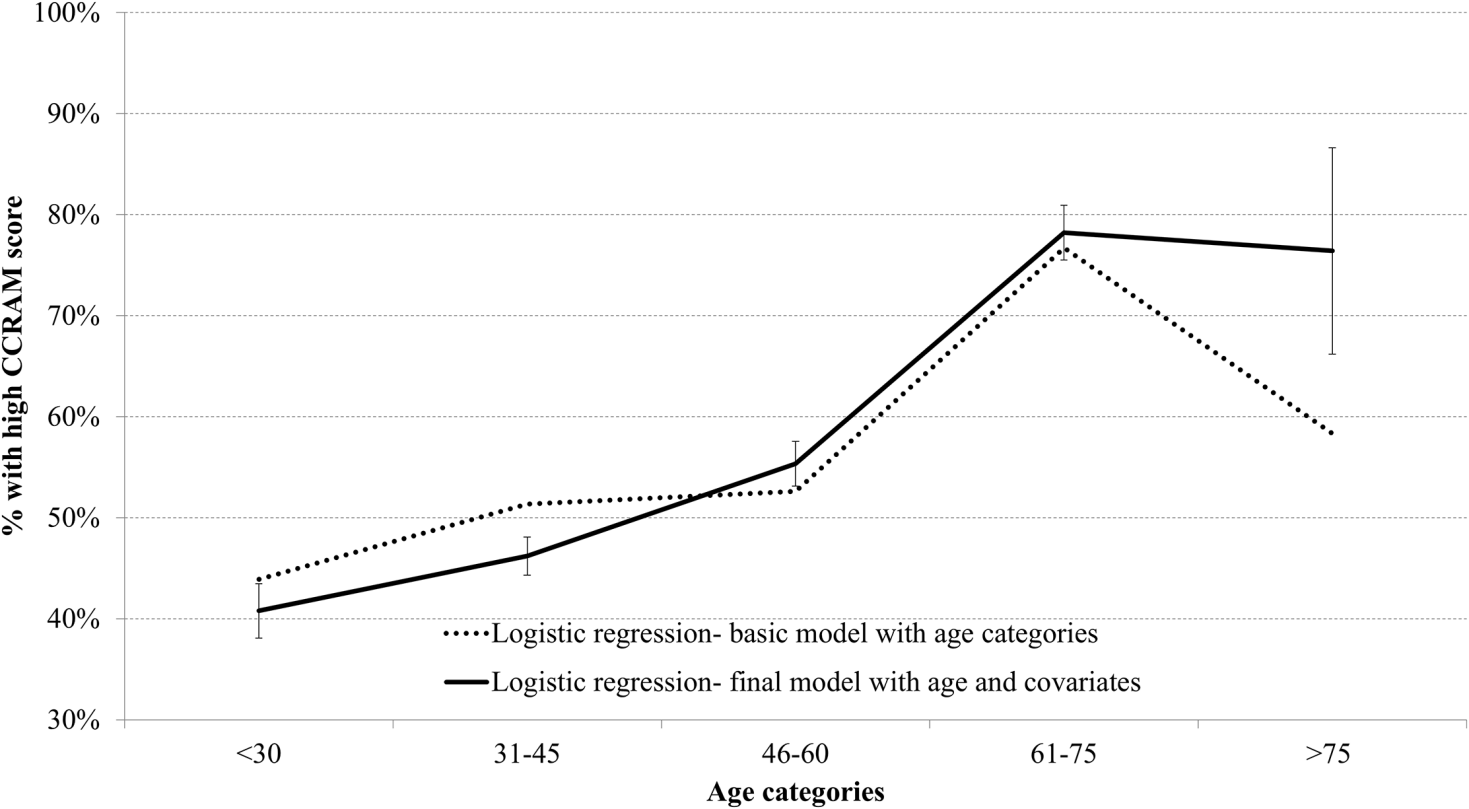
Probability of high resilience score by age categories as predicted by logistic regression models.

A logistic regression modeled high CCRAM score (mean score with the range 4–5) vs. low score (mean within the range 1–2.99). Initially the model only included age in the five categories mentioned above. Ages 31–45 were taken as the reference group. Over all, age had a significant association with CCRAM, and the age category of 61–75 years had an odds ratio of 3.12 (95% CI 1.66–5.86) with reference to age 31–45 (p < 0.001). After adjustment for study covariates (gender, marital status, children at home, disability, religion, travel time to work, education level, self-reported income level, belonging to a Community Emergency Response Team (CERT), and community type), this relationship had an even higher OR– 4.32 (95% CI 1.25–14.99), p = 0.021, for the same age category. The predicted probabilities of the two models over age are presented in Fig 3. The two models show a similar upward trend until the age category 61–75 years; thereafter the covariates adjustment moderates the downward trend at late age at >75 years. Table 2 presents the regression coefficients of the final model of logistic regression, which includes age categories and study covariates.

**Table 2:** Variables associated with community resilience score in the final model of logistic regression.

Variables	Odds Ratio	P-value	95% CI for OR	
			Lower	Upper
Age				
<30	1.18	0.739	0.438	3.199
31–45	1*			
46–60	1.22	0.565	0.616	2.433
61–75	4.32	0.021	1.246	14.994
>75	4.05	0.481	0.083	197.555
Gender				
Female	1			
Male	0.42	0.006	0.225	0.783
Family status				
In a permanent relationship	1			
Not in a permanent relationship	0.35	0.016	0.145	0.823
Child at home				
Yes	1			
No	1.48	0.341	0.661	3.309
Physical or mental disability				
No	1			
Yes	0.21	0.007	0.065	0.649
Education				
Non-academic	1			
Academic	0.46	0.017	0.244	0.869
Religion				
Secular	1			
Religious	1.65	0.165	0.814	3.332
Travel time to work				
<30 min	1			
> 30 min	0.64	0.277	0.281	1.439
Income				
Average	1			
Lower than average	0.95	0.895	0.457	1.982
Higher than average	0.36	0.008	0.169	0.764
CERT volunteering				
No	1			
Yes	2.78	0.042	1.036	7.487
Community Type				
Mid-size town	1			
Small communities	17.18	0.000	8.165	36.164

Note: -2 log likelihood = 272.800 (df = 15). Chi-square p<0.001.

*OR = 1 indicates the reference group.

Although the age category ‘61–75 years’ demonstrates on average higher CCRAM scores, it is unclear which cofactor explains this. For a further exploration of the covariates distribution over age, see Table 3, which lists conflicting evidence of high rates for both a risk factor (disability, p = 0.007, OR = 0.20) and a protective factor (community type, p < 0.001, OR = 17.2). Study covariates by the five age categories are presented in Table 3.

**Table 3:** Characteristics of socio-demographic variables by age categories.

Variables	<30 n = 179	31–45 n = 290	46–60 n = 249	61–75 n = 136	>75 n = 23
CCRAM score: mean, SE	3.41, 0.05	3.45, 0.04	3.50, 0.05	3.80, 0.06	3.78, 0.15
Female, %	55	58	59^D^	44	52
Not in permanent relationships, %	71^B^^C^^D^	16	19	13	50^B^^C^^D^
No child at home, %	66^B^^C^	10	22^B^	76^B^^C^	91^B^^C^
Unemployed %	28^B^^C^	8	11	35^B^^C^	70^A^^B^^C^^D^
With disability, %	5	6	14 ^A^^B^	34 ^A^^B^^C^	78 ^A^^B^^C^^D^
Education: academic, %	29	50 ^A^	42	48^A^	22
Religion: secular, %	66	54	55	69^B^	48
Income: less than average, %	39	31	32	35	65^B^^C^
CERT volunteering, %	5	12^A^	19^A^^D^	9	-
Community type: mid-size towns, %	60^D^	57 ^D^	54 ^D^	35	39

Note: Pairwise comparison with chi-square < 0.05 compared to age:

^A^ = <30 years,

^B^ = 31–45 years,

^C^ = 46–60 years,

^D^ = 61–75 years.

### Sensitivity analysis

**Fig 4:**
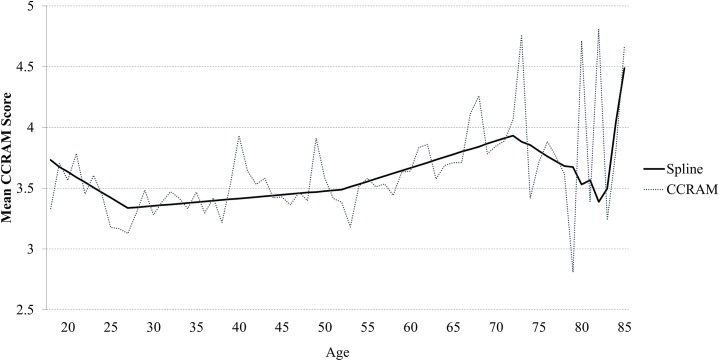
Mean CCRAM score by age categories and by selected characteristics.

The age trend of the CCRAM score was examined in subgroups with different characteristics. Fig 4 demonstrates that the trend persisted in all subgroups, with the CCRAM mean score increasing at 61–75 years for all subsets: men and women; presence of health disability; community type.

## Discussion

This study examined the association between the participants' age and community resilience scores as measured by CCRAM [6]. Using spline regression to explore resiliency trends over the life span has important advantages. First, it enables flexible modeling of the curve with no constraints on the prior shape as required by the polynomial fit, thus providing identification of the ages at which trend changes occur [31].

The study identified a significant rise in community resiliency scores at the later ages of 61–75 years as compared with younger age bands. Throughout this project, we used the full data set for analysis. However, in the regression modeling we found that the middle population may mask associations. We therefore present the significant contrast between the lower and upper scores, which is indicative of non-linearities in the association. After adjustment for study covariates (see Fig 3), the results reinforce the findings from the preliminary analysis and overcome existing sampling gaps. Examination of the factors affecting the community resilience score (presented in Table 2) reveals that the most influential is residence in a small community (OR = 17.2, p < 0.001). However, the resilience score obtained in the sensitivity analysis demonstrates that respondents living in mid-size towns behave differently from those living in small communities. While both show a tendency toward increased community resilience scores in the later decades of life, this increase is much more prominent in mid-size communities.

In a way this is puzzling, since the general perception of elders places them among those most vulnerable to the direct impact of disasters, especially as compared with the middle aged population [17]. Studies have noted the negative impact of exposure of older adults to disasters on somatic symptoms and medical and psychological co-morbidities [32]. Similarly, in response plans for emergencies, older adults are considered a sub-population with special needs [33], [34].

However, a different approach is described by Rabinovici [35] in his book "The six ages of man." Rabinovici suggests that old age is defined not by chronology but rather by the accumulation of various components, among which the internal perception of the individual is the most important. Lahad [36], supporting this approach, observes that while a certain proportion of the elderly have special needs and show a decline in objective characteristics such as response time and swiftness of motion, some features of old age are an asset and a resource [36]. In particular he mentions life experience, the fact that elders are free of most commitments and looking for something to do, their skills, and their need to feel meaningful and to be with others.

Research on ageing also reveals increasing scores in the later years of life in related fields. Jeste et al. [37] addressed this issue among participants suffering from schizophrenia and argued that, contrary to the tendency to consider ageing as a homogeneous process, the decline in cognitive functioning and physical health among such subjects contrasts sharply with the improvement in psychosocial functioning and subjective quality of life. Stone et al. [38] found a U shape distribution of wellbeing in relation to participants' ages, with a tendency to increased wellbeing in later life. Some studies have sought to find explanations for this seeming paradox. Some focused on the neurobiological processes that occur with aging [39], [40], while others found that social factors, psychological aspects and life-style–but not somatic co-morbidities–were relevant determinants of late life satisfaction [41]. Contrary to the findings of Stone et al. [38], Wunder et al. [31] have shown in a longitudinal study that there is another turning point of decline in the wellbeing distribution, a shift which was overlooked owing to the forced shape of the fitted polynomial in Stone's research. Wunder's findings [31] are similar to those in the current study. Frijters & Beatton [42] observed that in European and Australian databases there is a ‘happiness peak’ around the age of 70, with decrease in happiness as people grow old.

Schaie et al. [43] studied factors affecting cognitive functioning. According to them [43], continuity of performance is maintained until a drop off point around the age of 75 with the emergence of chronic disease and disability. In short, a variety of factors have contributed to the prolongation of both overall life expectancy and what has been termed a “useful” life expectancy relatively undiminished by illness or disability. All these findings provide further evidence of the similarity found between our curve of community resilience scores and other indicators [42],[43].

It is becoming increasingly common in various domains of research on the elderly to regard ageing as comprised of two stages. Ryff et al. [44] found that among studies which measured self-reported health, the earlier period of aging indicated, via social comparison processes, that later life physical health is associated with psychological well-being.

In a paper presented at the European parliament by Lahad and Fanaras [45] regarding the impact of the economic crisis in Greece, the authors report that persons aged 55 and above felt that they were faring better than in the past, in contrast to younger people, who felt worse. One explanation they offer is that the older generation went through so many crises in the contemporary history of Greece that the recent one is not so frightening for them [45].

Community resilience is not a collection of community members coping individually with adversity. Pfefferbaum et al. [46] emphasize that community resilience is "the ability of community members to take deliberate, purposeful, and collective action to alleviate the detrimental effects of adverse events." This approach may shed light on the ability of elders to contribute their community leadership experience, over and above their ability to cope personally with various challenges. These findings support the conclusions of Charles [47], namely that older adults develop strategies that mitigate negative emotions more efficiently than younger adults. Furthermore, it has been shown that retirees tend to expand their civic activities such as volunteering. They become more socially involved after retirement and, as such, they can be viewed as a valuable resource for society [48].

These findings could be useful in two ways: first, for identifying dimensions that assist the ageing population; and second, for seeking resources capable of enriching and empowering the community at large. Community resilience is important in routine life as well as during times of crisis [1], [2], [8]. However, Maxwell et al. [49] argue that the local community assumes particular importance in protracted crises. At such junctures in the community's lifecycle, it is crucial to identify resources that improve the community's collective capacity, enabling the development of community abilities to cope with changes or unexpected events. Nelson [4] argues that the perspective of resiliency can stimulate thinking about ways of radically reorganizing systems to meet needs and goals. Nelson suggests that the concepts of resiliency and adaptation are integrally intertwined with human values and goals; thus if the ageing population becomes a source of strength, this becomes one of the steps to achieve the resiliency goal.

### Limitation

This paper presents results of a cross-sectional study. Longitudinal studies dealing with the association between ageing population and the resiliency of their community could shed more light on issues identified here.

## Conclusion

This study revealed an increase in community resilience scores among the ageing population. A search for data on trends of different scores over the lifespan reveals similar trends in other indices. The present study suggests that the increase in life expectancy and the growing proportion of elders in the population implies something more than growing medical needs and years of dependency. The results presented above shows that older people have a different perception of their communities' resiliency, reflected in higher community resilience scores over all CCRAM factors. Thus elders are a potential resource for their community. Based on our findings, it is suggested that the later part of life be visualized as comprising two subgroups: the first consists of persons up to 75 years of age, where, as we show, the majority of the population feel resilient or at the 'happiness peak’ [42],[43]; and the second consists of persons above 75 years of age. This view, which has recently been suggested by authors from other disciplines as well [42–44], could result in a revision of decision makers’ attitudes towards the elder population. It has been shown that the contribution of this age group to a community in crisis can be based on their functionality rather than their age. It is our suggestion that, in conjunction with the promotion of relevant response plans suited to older adults’ needs, the unique input of this sub-population to the general community in emergencies be considered as a positive asset. This population can be included in community enhancement programs that utilize their resources and experience.

## Supporting Information

S1 Table Distribution of scores for individual CCRAM questions. (DOCX)

S2 Table Distribution of scores for CCRAM factors. (DOCX)
