# Supplementary material for: Community Resilience throughout the Lifespan – The Potential Contribution of Healthy Elders
Source: PLoS One. 2016 Feb 4;11(2):e0148125. doi: 10.1371/journal.pone.0148125 (PMC4741520; doi:10.1371/journal.pone.0148125)
Supplement: S1 Table — (DOCX) [file pone.0148125.s001.docx]

**S1 Table. Distribution of scores for individual CCRAM questions, n=885**

| No. | Phrase | Min | Max | Mean | SD |
| --- | --- | --- | --- | --- | --- |
| 1 | The municipal authority functions well. | 1 | 5 | 3.33 | 1.199 |
| 2 | There is mutual assistance and people care for one another. | 1 | 5 | 3.84 | 1.075 |
| 3 | My community is prepared for an emergency situation. | 1 | 5 | 3.15 | 1.117 |
| 4 | I am proud to tell others where I live. | 1 | 5 | 4.31 | .989 |
| 5 | Good relationships exist between various groups. | 1 | 5 | 3.47 | 0.983 |
| 6 | I trust the local decision makers | 1 | 5 | 2.93 | 1.245 |
| 7 | I can count on people in my community to help me in a crisis situation. | 1 | 5 | 3.90 | 1.106 |
| 8 | Residents are aware of their roles in an emergency situation. | 1 | 5 | 2.99 | 1.253 |
| 9 | I have a sense of belonging to my community. | 1 | 5 | 4.16 | 1.011 |
| 10 | Residents in my community trust each other. | 1 | 5 | 3.46 | 0.999 |
| 11 | In my community, Appropriate attention is given to the needs of children. | 1 | 5 | 3.44 | 1.125 |
| 12 | In my community, There are people who can help to cope with an emergency situation. | 1 | 5 | 3.97 | 1.015 |
| 13 | There are sufficient facilities for public protection (e.g. shelters, etc.) in my community | 1 | 5 | 3.13 | 1.238 |
| 14 | I remain in my community for ideological reasons. | 1 | 5 | 3.19 | 1.438 |
| 15 | I have faith in my mayor's ability to lead the transfer from routine to emergency management. | 1 | 5 | 3.07 | 1.221 |
| 16 | I have faith in my community's ability to overcome an emergency situation. | 1 | 5 | 3.94 | 1.026 |
| 17 | My family and I are acquainted with the emergency system in my town (to be activated in times of emergency). | 1 | 5 | 2.99 | 1.327 |
| 18 | I would be sorry to leave the town where I reside. | 1 | 5 | 4.04 | 1.287 |
| 19 | The municipal authorities fairly provide services | 1 | 5 | 3.15 | 1.172 |
| 20 | The residents are greatly involved in the community's activities. | 1 | 5 | 3.42 | 1.079 |
| 21 | The residents of my community will continue to receive Municipal services even in an emergency situation. | 1 | 5 | 3.33 | 1.091 |
| 22 | I feel safe in my place of residence. | 1 | 5 | 4.04 | 1.025 |
| 23 | The Health services in my town will continue to function appropriately in an emergency situation. | 1 | 5 | 3.56 | 1.147 |
| 24 | The information I receive from the municipal authority during emergency situations fulfill my needs. | 1 | 5 | 3.28 | 1.148 |
| 25 | Many of my neighbors are my friends. | 1 | 5 | 3.58 | 1.223 |
| 26 | I intend to leave my place of residence in an emergency. | 1 | 5 | 3.77 | 1.421 |
| 28 | Officials in my place of residence demonstrate leadership abilities. | 1 | 5 | 3.15 | 1.196 |
