# Supplementary material for: Community Resilience throughout the Lifespan – The Potential Contribution of Healthy Elders
Source: PLoS One. 2016 Feb 4;11(2):e0148125. doi: 10.1371/journal.pone.0148125 (PMC4741520; doi:10.1371/journal.pone.0148125)
Supplement: S2 Table — (DOCX) [file pone.0148125.s002.docx]

**S2 Table. Distribution of scores for CCRAM factors, n=885**

| CCRAM factor | Min | Max | Mean | SD |
| --- | --- | --- | --- | --- |
| Leadership | 1 | 5 | 3.21 | 1.002 |
| Collective efficacy | 1 | 5 | 3.82 | 0.832 |
| Preparedness | 1 | 5 | 3.06 | 1.025 |
| Place attachment | 1 | 5 | 3.93 | 0.918 |
| Social trust | 1 | 5 | 3.47 | 0.927 |
